# Bis{2-[2,5-bis­(pyridin-2-yl)-1*H*-imidazol-4-yl]pyridinium} tetra­cyanidoplatinate(II) tetra­hydrate

**DOI:** 10.1107/S1600536813011665

**Published:** 2013-05-04

**Authors:** Raquel Gámez-Heredia, Rosa E. Navarro, Herbert Höpfl, Adriana Cruz-Enríquez, José J. Campos-Gaxiola

**Affiliations:** a Departamento de Investigación en Polimeros y Materiales, Universidad de Sonora, Rosales y Blvd. Luis Encinas S/N, Col. Centro, Edificio 3G, CP 83000, Hermosillo, Sonora, México; b Centro de Investigaciones Quimicas, Universidad Autónoma del Estado de Morelos, Av. Universidad 1001, CP 62210, Cuernavaca, Morelos, México; c Facultad de Ingenieria Mochis, Universidad Autónoma de Sinaloa, Fuente Poseidon y Prol. A. Flores S/N, CP 81223, C.U. Los Mochis, Sinaloa, México

## Abstract

The asymmetric unit of the title hydrated complex salt, (C_18_H_14_N_5_)_2_[Pt(CN)_4_]·4H_2_O, consists of one 2-[2,5-bis­(pyridin-2-yl)-1*H*-imidazol-4-yl]pyridinium cation, half a tetra­cyanidoplatinate(II) dianion, which is located about a crystallographic inversion center, and two water mol­ecules of crystallization. The Pt^II^ atom has a square-planar coordination environment, with Pt—C_CN_ distances of 1.992 (4) and 2.000 (4) Å. In the cation, there is an N—H⋯N hydrogen bond linking adjacent pyridinium and pyridine rings in positions 4 and 5. Despite this, the organic component is non-planar, as shown by the dihedral angles of 10.3 (2), 6.60 (19) and 15.66 (18)° between the planes of the central imidazole ring and the pyridine/pyridinium substituents in the 2-, 4- and 5-positions. In the crystal, cations and anions are linked *via* O—H⋯O, O—H⋯N and N—H⋯O hydrogen bonds, forming a three-dimensional network. Additional π–π, C—H⋯O and C—H⋯N contacts provide stabilization to the crystal lattice.

## Related literature

For the structural, magnetic, optical and electrical properties of hydrogen-bonded inorganic–organic hybrid materials, see: Anastassiadou *et al.* (2000 ▶); Crawford *et al.* (2004 ▶); Dechambenoit *et al.* (2006 ▶); Du *et al.* (2013 ▶); Lebeau & Innocenzi (2011 ▶); Maynard & Sykora (2008 ▶); Pardo *et al.* (2011 ▶); Sanchez *et al.* (2005 ▶); Wang *et al.* (2010 ▶); Yao *et al.* (2010 ▶).
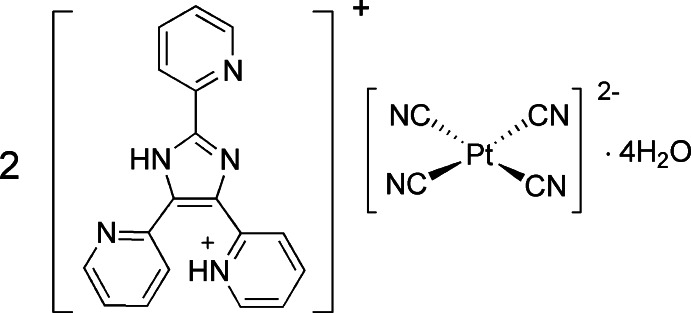


## Experimental

### 

#### Crystal data

(C_18_H_14_N_5_)_2_[Pt(CN)_4_]·4H_2_O
*M* *_r_* = 971.92
Monoclinic, 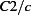

*a* = 20.958 (5) Å
*b* = 12.048 (3) Å
*c* = 15.403 (3) Å
β = 95.483 (3)°
*V* = 3871.6 (14) Å^3^
*Z* = 4
Mo *K*α radiation
μ = 3.69 mm^−1^
*T* = 100 K
0.50 × 0.34 × 0.28 mm

#### Data collection

Bruker SMART CCD area detector diffractometer
Absorption correction: multi-scan (*SADABS*; Sheldrick, 1996 ▶) *T*_min_ = 0.26, *T*_max_ = 0.43
17095 measured reflections
3418 independent reflections
2726 reflections with *I* > 2σ(*I*)
*R*_int_ = 0.066

#### Refinement

*R*[*F* ^2^ > 2σ(*F* ^2^)] = 0.032
*wR*(*F* ^2^) = 0.078
*S* = 1.00
3418 reflections
286 parameters
8 restraints
H atoms treated by a mixture of independent and constrained refinement
Δρ_max_ = 3.10 e Å^−3^
Δρ_min_ = −2.27 e Å^−3^

### 

Data collection: *SMART* (Bruker, 2001 ▶); cell refinement: *SAINT-Plus* (Bruker, 2001 ▶); data reduction: *SAINT-Plus*; program(s) used to solve structure: *SHELXS97* (Sheldrick, 2008 ▶); program(s) used to refine structure: *SHELXL97* (Sheldrick, 2008 ▶); molecular graphics: *ORTEP-3 for Windows* (Farrugia, 2012 ▶) and *Mercury* (Macrae *et al.*, 2008 ▶); software used to prepare material for publication: *SHELXTL* (Sheldrick, 2008 ▶) and *publCIF* (Westrip, 2010 ▶).

## Supplementary Material

Crystal structure: contains datablock(s) I, global. DOI: 10.1107/S1600536813011665/su2593sup1.cif

Structure factors: contains datablock(s) I. DOI: 10.1107/S1600536813011665/su2593Isup2.hkl

Additional supplementary materials:  crystallographic information; 3D view; checkCIF report

## Figures and Tables

**Table 1:** Hydrogen-bond geometry (Å, °)

*D*—H⋯*A*	*D*—H	H⋯*A*	*D*⋯*A*	*D*—H⋯*A*
N5—H5′⋯N4	0.84	1.83	2.609 (4)	154
N1—H1′⋯O31^i^	0.84	1.94	2.771 (4)	169
O31—H31*B*⋯O32^ii^	0.84	2.02	2.778 (4)	151
O32—H32*A*⋯N6^iii^	0.84	2.12	2.937 (4)	164
O31—H31*A*⋯N3^iv^	0.84	2.05	2.822 (5)	152
O32—H32*B*⋯N7^v^	0.84	2.02	2.854 (5)	170
C6—H6⋯O32^vi^	0.95	2.68	3.564 (5)	155
C17—H17⋯O32^vii^	0.95	2.88	3.459 (5)	120
C18—H18⋯O32^vii^	0.95	2.70	3.375 (5)	129
C18—H18⋯N6^viii^	0.95	2.50	3.406 (5)	161

Symmetry codes: (i) 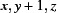; (ii) 
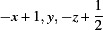
; (iii) 
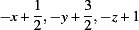
; (iv) 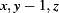; (v) 
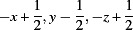
; (vi) 
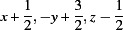
; (vii) 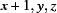; (viii) 
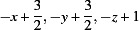
.

**Fig. 1.**
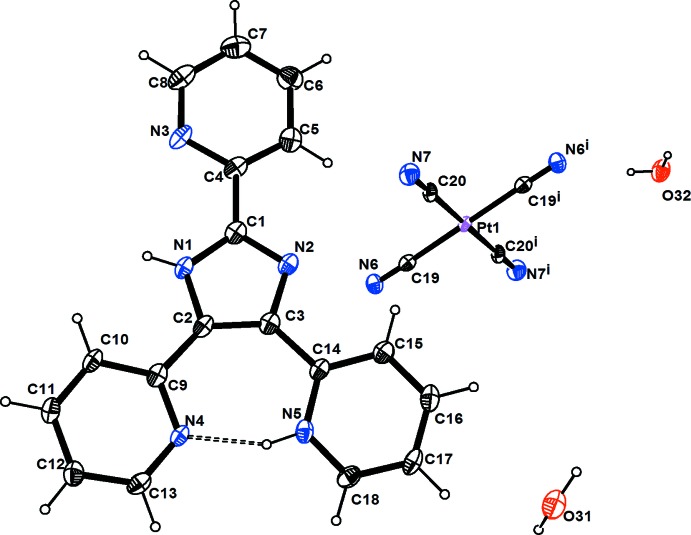
The molecular structure of the title compound, with atom labelling. Displacement ellipsoids are drawn at the 50% probability level [symmetry code: (i) -x + 1/2, -y + 3/2, -z + 1].

**Fig. 2.**
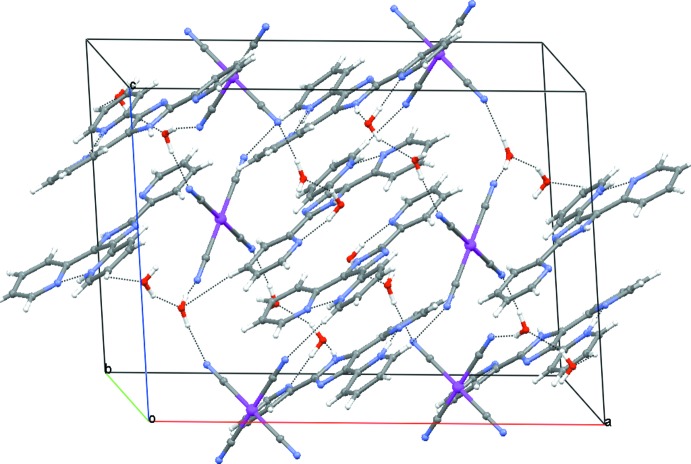
A view along the b axis of the crystal packing of the title compound, showing the O—H···O, O—H···N, N—H···O, C—H···O and C—H···N hydrogen bonded three-dimensional supramolecular network (hydrogen bonds are shown as dashed lines; see Table 1 for details).

## supplementary crystallographic information

### Comment

Hydrogen bond based inorganic–organic hybrid materials are receiving continuous interest because of their structural, magnetic, optical and electrical properties (Sanchez *et al.*, 2005; Yao *et al.*, 2010; Wang *et al.*, 2010; Lebeau *et al.*, 2011; Pardo *et al.*, 2011; Du *et al.*, 2013). An interesting approach for the preparation of such materials consists in the utilization of supramolecular synthons capable of forming O—H···O, O—H···N and N—H···O hydrogen bonds, through which organic cations and anionic metal complexes are linked to form crystalline inorganic–organic hybrid solids (Crawford *et al.*, 2004; Dechambenoit *et al.*, 2006; Maynard & Sykora, 2008). As a further contribution to this filed of research we report herein on the synthesis and crystal structure of the title compound.

The asymmetric unit of the title compound consists of one organic cation of the composition [(C_18_H_14_N_5_)]^+^, which is located in a general position, half of an independent [Pt(CN)_4_]^2-^ anion, which is located on a crystallographic inversion center, and two water molecules of crystallization (Fig. 1). The Pt atom has a square-planar coordination environment with Pt—C(cyanido) distances ranging from 1.992 (4) to 2.000 (4) Å. Although two of the three pyridine substituents are linked by an intramolecular N5^+^—H5'···N4 hydrogen bond (Table 1), the organic component is essentially nonplanar, as shown by the dihedral angles of 10.3 (2), 6.60 (19) and 15.66 (18)°, respectively, formed between the central imidazole ring plane and the pyridine substituents in the 2-, 4- and 5-positions.

In the crystal, the cations and anions are linked by O—H···O, O—H···N and N—H···O hydrogen bonds forming a three-dimensional network (Table 1 and Fig. 2). Within the network, the organic cations are stacked through a series of π—π interactions involving the imidazole and pyridinium rings [*Cg*1···*Cg*1^i^ distance = 3.359 (2) Å; *Cg*1···*Cg*2^ii^ distance = 3.559 (2) Å; *Cg*3···*Cg*3^iii^ distance = 4.023 (3) Å; *Cg*2···*Cg*3^i^ distance = 3.929 (2) Å; *Cg*1= N1/C1/N2/C3/C2; *Cg*2 = N4/C9-C13 and *Cg*3 = N3/C4—C8; symmetry codes: (i) –x+1, -*y+1*, -*z*; (ii) –x+1, *y*, 1- *z+1/2*; (iii)-*x+1/2*, - *y+3/2*, -*z*]. Additionally, there are a number of C—H···O and C—H···N contacts present, thus providing additional stabilization to the crystal lattice (Table 1 and Fig. 2).

### Experimental

The organic component in the title compound, 2-(2,5-di(pyridin-2-yl)-1*H*-imidazol-4-yl)pyridine, was synthesized according to a previously reported procedure (Anastassiadou *et al.*, 2000). Single crystals of the platinum complex were prepared at room temperature by slow diffusion of a CH_3_CN/H_2_O (2 ml, 1:1 *v*/*v*) solution containing both Eu(NO_3_)_3_^ . ^6H_2_O (0.034 g, 0.08 mmol) and K_2_Pt(CN)_4_ (0.030 g, 0.08 mmol) into a CH_2_Cl_2_ (2 ml) solution of 2-(2,5-di(pyridin-2-yl)-1*H*-imidazol-4-yl)pyridine (0.024 g, 0.08 mmol). After two weeks, pale yellow crystals were obtained [Yield: 40%]. IR and TGA details are given in the archived CIF.

### Refinement

The C-bound H atoms were positioned geometrically and refined as riding atoms [aryl C—H = 0.95 Å and *U*_iso_(H) = 1.2*U*_eq_(C)]. The pyridinium N—H^+^ and water H atoms were located in difference Fourier maps. They were refined with distance restraints of 0.840 (1)Å, with *U*_iso_(H) = 1.5*U*_eq_(O, N). For the refinement of the water molecules a constraint has also been employed for the H—O—H bond angles (DANG = 1.35 (1) Å). In the final difference Fourier map a large residual density peak, 3.10 eÅ^-3^, and hole, -2.26 eÅ^-3^, were found near the Pt atom.

### Crystal data

**Table:**

(C_18_H_14_N_5_)_2_[Pt(CN)_4_]·4H_2_O	*F*(000) = 1936
*M**_r_* = 971.92	*D*_x_ = 1.667 Mg m^−^^3^
Monoclinic, *C*2/*c*	Mo *K*α radiation, λ = 0.71073 Å
Hall symbol: -C 2yc	Cell parameters from 9440 reflections
*a* = 20.958 (5) Å	θ = 2.3–27.7°
*b* = 12.048 (3) Å	µ = 3.69 mm^−^^1^
*c* = 15.403 (3) Å	*T* = 100 K
β = 95.483 (3)°	Rectangular prism, yellow
*V* = 3871.6 (14) Å^3^	0.50 × 0.34 × 0.28 mm
*Z* = 4	

### Data collection

**Table:**

Bruker SMART CCD area detector diffractometer	3418 independent reflections
Radiation source: fine-focus sealed tube	2726 reflections with *I* > 2σ(*I*)
Graphite monochromator	*R*_int_ = 0.066
phi and ω scans	θ_max_ = 25.0°, θ_min_ = 2.0°
Absorption correction: multi-scan (*SADABS*; Sheldrick, 1996)	*h* = −24→24
*T*_min_ = 0.26, *T*_max_ = 0.43	*k* = −14→14
17095 measured reflections	*l* = −18→18

### Refinement

**Table:**

Refinement on *F*^2^	Primary atom site location: structure-invariant direct methods
Least-squares matrix: full	Secondary atom site location: difference Fourier map
*R*[*F*^2^ > 2σ(*F*^2^)] = 0.032	Hydrogen site location: inferred from neighbouring sites
*wR*(*F*^2^) = 0.078	H atoms treated by a mixture of independent and constrained refinement
*S* = 1.00	*w* = 1/[σ^2^(*F*_o_^2^) + (0.0497*P*)^2^] where *P* = (*F*_o_^2^ + 2*F*_c_^2^)/3
3418 reflections	(Δ/σ)_max_ < 0.001
286 parameters	Δρ_max_ = 3.10 e Å^−^^3^
8 restraints	Δρ_min_ = −2.27 e Å^−^^3^

### Special details

**Table:**

Experimental. IR and TGA details for the title compound: IR(KBr, cm^-1^): 3406, 3385, 3268, 3100, 2129, 1661, 1596, 1501, 1447, 1235, 1139, 1096, 777, 703. TGA: Calcd. for 4H_2_O: 7.42. Found: 7.69% (298 - 385 K).
Geometry. All e.s.d.'s (except the e.s.d. in the dihedral angle between two l.s. planes) are estimated using the full covariance matrix. The cell e.s.d.'s are taken into account individually in the estimation of e.s.d.'s in distances, angles and torsion angles; correlations between e.s.d.'s in cell parameters are only used when they are defined by crystal symmetry. An approximate (isotropic) treatment of cell e.s.d.'s is used for estimating e.s.d.'s involving l.s. planes.
Refinement. Refinement of *F*^2^ against ALL reflections. The weighted *R*-factor *wR* and goodness of fit *S* are based on *F*^2^, conventional *R*-factors *R* are based on *F*, with *F* set to zero for negative *F*^2^. The threshold expression of *F*^2^ > σ(*F*^2^) is used only for calculating *R*-factors(gt) *etc*. and is not relevant to the choice of reflections for refinement. *R*-factors based on *F*^2^ are statistically about twice as large as those based on *F*, and *R*- factors based on ALL data will be even larger.

### Fractional atomic coordinates and isotropic or equivalent isotropic displacement parameters (Å^2^)

**Table:**

	*x*	*y*	*z*	*U*_iso_*/*U*_eq_	
Pt1	0.2500	0.7500	0.5000	0.01672 (10)	
N1	0.96962 (15)	1.0885 (2)	0.10252 (19)	0.0173 (7)	
H1'	0.973 (2)	1.1564 (9)	0.114 (3)	0.026*	
N2	0.92075 (14)	0.9356 (2)	0.05054 (18)	0.0179 (7)	
N3	0.87601 (18)	1.2211 (3)	−0.0001 (2)	0.0226 (8)	
N4	1.09913 (14)	0.9336 (2)	0.22311 (18)	0.0171 (7)	
N5	1.03998 (19)	0.7543 (2)	0.1645 (2)	0.0189 (8)	
H5'	1.0631 (17)	0.798 (3)	0.196 (2)	0.028*	
N6	0.31880 (15)	0.8613 (3)	0.6676 (2)	0.0256 (8)	
N7	0.32877 (17)	0.9085 (3)	0.3882 (2)	0.0320 (8)	
C1	0.91839 (17)	1.0457 (3)	0.0535 (2)	0.0181 (8)	
C2	1.00799 (17)	1.0022 (3)	0.1331 (2)	0.0168 (8)	
C3	0.97619 (18)	0.9069 (3)	0.1007 (2)	0.0170 (9)	
C4	0.86797 (17)	1.1094 (3)	0.0042 (2)	0.0188 (8)	
C5	0.81594 (17)	1.0555 (3)	−0.0383 (2)	0.0238 (9)	
H5	0.8109	0.9777	−0.0321	0.029*	
C6	0.77141 (19)	1.1165 (3)	−0.0900 (2)	0.0286 (10)	
H6	0.7355	1.0812	−0.1206	0.034*	
C7	0.7798 (3)	1.2285 (4)	−0.0962 (3)	0.0325 (11)	
H7	0.7502	1.2718	−0.1325	0.039*	
C8	0.8316 (2)	1.2785 (4)	−0.0496 (3)	0.0290 (10)	
H8	0.8358	1.3568	−0.0528	0.035*	
C9	1.07022 (17)	1.0222 (3)	0.1828 (2)	0.0172 (8)	
C10	1.09906 (18)	1.1263 (3)	0.1866 (2)	0.0201 (8)	
H10	1.0780	1.1884	0.1588	0.024*	
C11	1.15875 (19)	1.1376 (3)	0.2316 (2)	0.0218 (9)	
H11	1.1794	1.2079	0.2344	0.026*	
C12	1.18881 (19)	1.0470 (3)	0.2727 (2)	0.0225 (9)	
H12	1.2301	1.0535	0.3037	0.027*	
C13	1.15672 (18)	0.9468 (3)	0.2671 (2)	0.0218 (9)	
H13	1.1765	0.8842	0.2959	0.026*	
C14	0.99006 (18)	0.7888 (3)	0.1090 (2)	0.0155 (8)	
C15	0.95298 (19)	0.7094 (3)	0.0622 (2)	0.0196 (9)	
H15	0.9177	0.7314	0.0226	0.023*	
C16	0.9678 (2)	0.5986 (3)	0.0734 (3)	0.0241 (9)	
H16	0.9424	0.5439	0.0420	0.029*	
C17	1.01988 (19)	0.5669 (3)	0.1306 (2)	0.0230 (9)	
H17	1.0306	0.4907	0.1386	0.028*	
C18	1.05545 (19)	0.6473 (3)	0.1752 (2)	0.0215 (9)	
H18	1.0914	0.6269	0.2141	0.026*	
C19	0.29365 (18)	0.8199 (3)	0.6075 (2)	0.0204 (9)	
C20	0.29937 (19)	0.8513 (3)	0.4291 (2)	0.0220 (9)	
O31	0.96490 (15)	0.3159 (2)	0.1280 (2)	0.0341 (7)	
H31A	0.9379 (13)	0.310 (4)	0.0842 (14)	0.051*	
H32A	0.1394 (19)	0.481 (2)	0.304 (2)	0.051*	
O32	0.12373 (15)	0.4241 (2)	0.27892 (18)	0.0324 (7)	
H31B	0.9448 (15)	0.334 (4)	0.1704 (15)	0.049*	
H32B	0.135 (2)	0.426 (3)	0.2280 (11)	0.049*	

### Atomic displacement parameters (Å^2^)

**Table:**

	*U*^11^	*U*^22^	*U*^33^	*U*^12^	*U*^13^	*U*^23^
Pt1	0.01960 (15)	0.01288 (15)	0.01807 (14)	0.00058 (8)	0.00385 (8)	−0.00070 (7)
N1	0.0203 (18)	0.0123 (16)	0.0202 (16)	0.0003 (15)	0.0066 (13)	−0.0003 (14)
N2	0.0202 (18)	0.0155 (17)	0.0191 (16)	−0.0003 (14)	0.0083 (13)	0.0013 (13)
N3	0.027 (2)	0.0138 (16)	0.029 (2)	0.0015 (16)	0.0114 (15)	0.0038 (15)
N4	0.0224 (18)	0.0111 (16)	0.0184 (16)	0.0006 (14)	0.0048 (13)	0.0000 (13)
N5	0.021 (2)	0.016 (2)	0.0201 (19)	−0.0058 (14)	0.0047 (14)	−0.0014 (13)
N6	0.026 (2)	0.0281 (19)	0.0228 (18)	−0.0027 (16)	0.0053 (15)	−0.0046 (15)
N7	0.035 (2)	0.031 (2)	0.031 (2)	−0.0065 (17)	0.0077 (16)	0.0025 (16)
C1	0.019 (2)	0.018 (2)	0.0183 (19)	0.0004 (17)	0.0099 (16)	0.0001 (16)
C2	0.022 (2)	0.013 (2)	0.0169 (19)	0.0014 (17)	0.0098 (15)	0.0005 (16)
C3	0.020 (2)	0.018 (2)	0.0152 (19)	−0.0019 (17)	0.0090 (16)	0.0016 (16)
C4	0.020 (2)	0.018 (2)	0.0200 (19)	0.0058 (17)	0.0117 (16)	0.0013 (16)
C5	0.023 (2)	0.024 (2)	0.025 (2)	−0.0002 (19)	0.0073 (17)	−0.0027 (17)
C6	0.026 (2)	0.030 (2)	0.030 (2)	0.006 (2)	0.0015 (17)	−0.0021 (19)
C7	0.033 (3)	0.034 (3)	0.030 (3)	0.011 (2)	0.002 (2)	0.006 (2)
C8	0.035 (3)	0.021 (2)	0.033 (3)	0.005 (2)	0.014 (2)	0.008 (2)
C9	0.022 (2)	0.016 (2)	0.0156 (19)	0.0004 (17)	0.0100 (15)	−0.0019 (15)
C10	0.028 (2)	0.0115 (19)	0.022 (2)	0.0001 (18)	0.0067 (16)	0.0003 (16)
C11	0.025 (2)	0.015 (2)	0.026 (2)	−0.0034 (18)	0.0045 (17)	−0.0038 (17)
C12	0.026 (2)	0.019 (2)	0.022 (2)	−0.0032 (18)	0.0025 (17)	−0.0033 (17)
C13	0.028 (2)	0.017 (2)	0.021 (2)	0.0022 (18)	0.0054 (17)	0.0052 (16)
C14	0.018 (2)	0.0142 (17)	0.016 (2)	−0.0009 (18)	0.0092 (16)	0.0017 (16)
C15	0.021 (2)	0.0181 (19)	0.020 (2)	0.0000 (19)	0.0057 (16)	0.0009 (17)
C16	0.029 (2)	0.019 (2)	0.025 (2)	−0.0070 (19)	0.0061 (18)	−0.0042 (17)
C17	0.028 (2)	0.012 (2)	0.030 (2)	−0.0029 (19)	0.0095 (18)	0.0008 (17)
C18	0.025 (2)	0.018 (2)	0.023 (2)	−0.0006 (18)	0.0058 (16)	0.0064 (17)
C19	0.024 (2)	0.019 (2)	0.020 (2)	0.0026 (18)	0.0069 (17)	0.0014 (17)
C20	0.025 (2)	0.019 (2)	0.022 (2)	−0.0011 (18)	0.0024 (17)	−0.0068 (17)
O31	0.0371 (19)	0.0219 (17)	0.0444 (19)	−0.0019 (15)	0.0096 (14)	−0.0049 (15)
O32	0.046 (2)	0.0254 (16)	0.0277 (16)	−0.0057 (15)	0.0115 (14)	−0.0006 (13)

### Geometric parameters (Å, º)

**Table:**

Pt1—C20	1.992 (4)	C6—C7	1.365 (5)
Pt1—C20^i^	1.992 (4)	C6—H6	0.9500
Pt1—C19	2.000 (4)	C7—C8	1.381 (7)
Pt1—C19^i^	2.000 (4)	C7—H7	0.9500
N1—C1	1.354 (5)	C8—H8	0.9500
N1—C2	1.370 (5)	C9—C10	1.391 (5)
N1—H1'	0.8400 (11)	C10—C11	1.378 (5)
N2—C1	1.328 (4)	C10—H10	0.9500
N2—C3	1.377 (5)	C11—C12	1.383 (5)
N3—C8	1.338 (6)	C11—H11	0.9500
N3—C4	1.358 (5)	C12—C13	1.381 (5)
N4—C13	1.336 (5)	C12—H12	0.9500
N4—C9	1.349 (4)	C13—H13	0.9500
N5—C18	1.336 (4)	C14—C15	1.391 (6)
N5—C14	1.351 (6)	C15—C16	1.377 (6)
N5—H5'	0.8400 (11)	C15—H15	0.9500
N6—C19	1.135 (5)	C16—C17	1.390 (6)
N7—C20	1.151 (4)	C16—H16	0.9500
C1—C4	1.459 (5)	C17—C18	1.367 (5)
C2—C3	1.396 (5)	C17—H17	0.9500
C2—C9	1.467 (5)	C18—H18	0.9500
C3—C14	1.455 (5)	O31—H31A	0.8401 (11)
C4—C5	1.380 (5)	O31—H31B	0.8400 (11)
C5—C6	1.379 (5)	O32—H32A	0.8401 (11)
C5—H5	0.9500	O32—H32B	0.8400 (11)
			
C20—Pt1—C20^i^	180.00 (17)	N3—C8—H8	118.6
C20—Pt1—C19	88.59 (15)	C7—C8—H8	118.6
C20^i^—Pt1—C19	91.41 (14)	N4—C9—C10	121.3 (3)
C20—Pt1—C19^i^	91.41 (14)	N4—C9—C2	116.6 (3)
C20^i^—Pt1—C19^i^	88.59 (15)	C10—C9—C2	122.1 (3)
C19—Pt1—C19^i^	180.000 (1)	C11—C10—C9	118.7 (3)
C1—N1—C2	108.2 (3)	C11—C10—H10	120.6
C1—N1—H1'	123 (3)	C9—C10—H10	120.6
C2—N1—H1'	129 (3)	C10—C11—C12	120.3 (4)
C1—N2—C3	105.3 (3)	C10—C11—H11	119.8
C8—N3—C4	117.2 (4)	C12—C11—H11	119.8
C13—N4—C9	118.9 (3)	C13—C12—C11	117.6 (4)
C18—N5—C14	122.6 (4)	C13—C12—H12	121.2
C18—N5—H5'	115 (3)	C11—C12—H12	121.2
C14—N5—H5'	123 (3)	N4—C13—C12	123.1 (3)
N2—C1—N1	111.6 (3)	N4—C13—H13	118.4
N2—C1—C4	122.3 (3)	C12—C13—H13	118.4
N1—C1—C4	125.9 (3)	N5—C14—C15	118.5 (4)
N1—C2—C3	104.9 (3)	N5—C14—C3	119.5 (4)
N1—C2—C9	121.2 (3)	C15—C14—C3	122.0 (4)
C3—C2—C9	133.8 (3)	C16—C15—C14	119.6 (4)
N2—C3—C2	110.0 (3)	C16—C15—H15	120.2
N2—C3—C14	116.5 (3)	C14—C15—H15	120.2
C2—C3—C14	133.5 (4)	C15—C16—C17	120.0 (4)
N3—C4—C5	122.6 (4)	C15—C16—H16	120.0
N3—C4—C1	117.4 (3)	C17—C16—H16	120.0
C5—C4—C1	120.0 (3)	C18—C17—C16	118.8 (4)
C6—C5—C4	118.9 (4)	C18—C17—H17	120.6
C6—C5—H5	120.5	C16—C17—H17	120.6
C4—C5—H5	120.5	N5—C18—C17	120.5 (4)
C7—C6—C5	118.8 (4)	N5—C18—H18	119.7
C7—C6—H6	120.6	C17—C18—H18	119.7
C5—C6—H6	120.6	N6—C19—Pt1	178.6 (3)
C6—C7—C8	119.6 (5)	N7—C20—Pt1	178.8 (3)
C6—C7—H7	120.2	H31A—O31—H31B	107.2 (12)
C8—C7—H7	120.2	H32A—O32—H32B	106.3 (12)
N3—C8—C7	122.7 (4)		
			
C3—N2—C1—N1	0.4 (4)	C13—N4—C9—C10	−0.4 (5)
C3—N2—C1—C4	176.7 (3)	C13—N4—C9—C2	178.1 (3)
C2—N1—C1—N2	0.4 (4)	N1—C2—C9—N4	168.6 (3)
C2—N1—C1—C4	−175.8 (3)	C3—C2—C9—N4	−17.0 (5)
C1—N1—C2—C3	−0.9 (4)	N1—C2—C9—C10	−12.9 (5)
C1—N1—C2—C9	175.0 (3)	C3—C2—C9—C10	161.5 (4)
C1—N2—C3—C2	−1.0 (4)	N4—C9—C10—C11	1.1 (5)
C1—N2—C3—C14	179.5 (3)	C2—C9—C10—C11	−177.4 (3)
N1—C2—C3—N2	1.2 (4)	C9—C10—C11—C12	−0.7 (5)
C9—C2—C3—N2	−173.9 (3)	C10—C11—C12—C13	−0.3 (5)
N1—C2—C3—C14	−179.4 (4)	C9—N4—C13—C12	−0.7 (5)
C9—C2—C3—C14	5.5 (7)	C11—C12—C13—N4	1.0 (5)
C8—N3—C4—C5	−1.4 (5)	C18—N5—C14—C15	0.8 (6)
C8—N3—C4—C1	176.1 (3)	C18—N5—C14—C3	−179.9 (3)
N2—C1—C4—N3	−168.9 (3)	N2—C3—C14—N5	−173.1 (3)
N1—C1—C4—N3	6.9 (5)	C2—C3—C14—N5	7.4 (6)
N2—C1—C4—C5	8.7 (5)	N2—C3—C14—C15	6.1 (5)
N1—C1—C4—C5	−175.5 (3)	C2—C3—C14—C15	−173.3 (4)
N3—C4—C5—C6	2.5 (5)	N5—C14—C15—C16	0.1 (5)
C1—C4—C5—C6	−175.0 (3)	C3—C14—C15—C16	−179.1 (4)
C4—C5—C6—C7	−1.0 (6)	C14—C15—C16—C17	−0.7 (5)
C5—C6—C7—C8	−1.4 (7)	C15—C16—C17—C18	0.3 (6)
C4—N3—C8—C7	−1.1 (6)	C14—N5—C18—C17	−1.2 (6)
C6—C7—C8—N3	2.6 (7)	C16—C17—C18—N5	0.6 (6)

Symmetry code: (i) −*x*+1/2, −*y*+3/2, −*z*+1.

### Hydrogen-bond geometry (Å, º)

**Table:**

*D*—H···*A*	*D*—H	H···*A*	*D*···*A*	*D*—H···*A*
N5—H5′···N4	0.84	1.83	2.609 (4)	154
N1—H1′···O31^ii^	0.84	1.94	2.771 (4)	169
O31—H31*B*···O32^iii^	0.84	2.02	2.778 (4)	151
O32—H32*A*···N6^i^	0.84	2.12	2.937 (4)	164
O31—H31*A*···N3^iv^	0.84	2.05	2.822 (5)	152
O32—H32*B*···N7^v^	0.84	2.02	2.854 (5)	170
C6—H6···O32^vi^	0.95	2.68	3.564 (5)	155
C17—H17···O32^vii^	0.95	2.88	3.459 (5)	120
C18—H18···O32^vii^	0.95	2.70	3.375 (5)	129
C18—H18···N6^viii^	0.95	2.50	3.406 (5)	161

Symmetry codes: (i) −*x*+1/2, −*y*+3/2, −*z*+1; (ii) *x*, *y*+1, *z*; (iii) −*x*+1, *y*, −*z*+1/2; (iv) *x*, *y*−1, *z*; (v) −*x*+1/2, *y*−1/2, −*z*+1/2; (vi) *x*+1/2, −*y*+3/2, *z*−1/2; (vii) *x*+1, *y*, *z*; (viii) −*x*+3/2, −*y*+3/2, −*z*+1.
